# Review of Low-Cost Photoacoustic Sensing and Imaging Based on Laser Diode and Light-Emitting Diode

**DOI:** 10.3390/s18072264

**Published:** 2018-07-13

**Authors:** Hongtao Zhong, Tingyang Duan, Hengrong Lan, Meng Zhou, Fei Gao

**Affiliations:** 1 Hybrid Imaging System Laboratory, School of Information Science and Technology, ShanghaiTech University, Shanghai 201210, China; zhonght@shanghaitech.edu.cn (H.Z.); duanty@shanghaitech.edu.cn (T.D.); lanhr@shanghaitech.edu.cn (H.L.); zhoumeng@shanghaitech.edu.cn (M.Z.); 2 Shanghai Advanced Research Institute, Chinese Academy of Sciences, Shanghai 201210, China

**Keywords:** photoacoustics imaging, sensing, low-cost laser source, laser diode

## Abstract

Photoacoustic tomography (PAT), a promising medical imaging method that combines optical and ultrasound techniques, has been developing for decades mostly in preclinical application. A recent trend is to utilize the economical laser source to develop a low-cost sensing and imaging system, which aims at an affordable solution in clinical application. These low-cost laser sources have different modulation modes such as pulsed modulation, continuous modulation and coded modulation to generate different profiles of PA signals in photoacoustic (PA) imaging. In this paper, we review the recent development of the photoacoustic sensing and imaging based on the economical laser sources such as laser diode (LD) and light-emitting diode (LED) in different kinds of modulation types, and discuss several representative methods to improve the performance of such imaging systems based on low-cost laser sources. Finally, some perspectives regarding the future development of portable PAT systems are discussed, followed by the conclusion.

## 1. Introduction

PAT is a kind of hybrid imaging modality, which employs the high imaging contrast of optical imaging and deep penetration of ultrasound imaging [1,2,3,4,5]. In PAT, the tissue is irradiated by the sufficient short-pulsed laser beam, and absorbs the energy partially, and then converts the absorbed energy into heat. The transient temperature elevation causes the expansion and contraction of the tissue and then produces the ultrasound wave, which will be further received by ultrasound transducers for image reconstruction [6,7,8,9,10,11,12]. PAT can be used in vivo for tumor detection, blood oxygenation mapping, animal or human organs imaging, etc. [13].

Conventionally, the light source usually comprises of a Q-switched Nd:YAG laser with nanosecond pulse duration and hundreds of mJ pulse energy, which is commonly used in different kinds of PAT imaging implementations, such as optical-resolution photoacoustic microscopy (OR-PAM), acoustic-resolution photoacoustic microscopy (AR-PAM) and photoacoustic computed tomography (PACT) [14,15,16,17,18,19]. From organelles to small animals or human organ imaging, and covering functional, metabolic and histologic imaging, the flash-lamp-pumped laser source can provide the sufficient pulse energy (several hundred mJ) and enough signal-to-noise ratio (SNR) of PA signals. The above-mentioned works are summarized in References [13,20,21,22]. However, this kind of laser source is widely used in the laboratory but is not economical and compact in clinical application since its high price and bulky size. Moreover, the repetition rate of most high-power laser sources is relatively low (~tens of Hz), which may limit the imaging speed when data averaging is required. Additionally, another type of laser source, diode pumped laser such as that mentioned in Reference [23], can operate in the repetition rate of 50 Hz with the 80 mJ per pulse.

In recent years, the low-cost and compact photoacoustic imaging system has been developing fast by utilizing laser diodes or light emitting diodes. Although the output peak energy of these semiconductor devices are low (nano-joule to micro-joule per pulse), it has the following advantages:

(1) The small sizes can reduce the volume of the imaging system significantly;
(2) Commercially available, which can be easily obtained from the industrial market;
(3) High repetition rate, up to tens of kilohertz, which can compensate for the low output energy to improve the SNR by averaging, which is promising to achieve real-time imaging;
(4) Does not require the active cooling system since the pulse duty cycle is less than 1%;
(5) The stability well meets the photoacoustic imaging system as the laser diode can maintain the constant output energy when working over 3000 h [24].

In addition to the above advantages, the ability of photoacoustic imaging by using these semiconductor devices has also been verified in many experiments such as those in References [24,25,26,27,28].

Because such semiconductor laser diodes can operate in high frequency with high-speed current driver, there are several modulation ways in signal excitation, such as pulsed modulation and continuous modulation. Pulsed modulation can be further divided into short-pulsed modulation based on LDs, short-pulse modulation based on LEDs, long-pulse modulation and coded excitation. Continuous modulation can be further divided into chirp modulation, sinusoidal modulation and hybrid modulation. Here, this review paper will discuss the recent development of low-cost photoacoustic sensing and imaging system categorized by different modulation ways. In the meantime, some signal enhancement methods to improve the SNR of PA imaging system will also be discussed.

## 2. Pulse Excitation

Using laser diode to generate short pulse is a very common method to excite the PA signals. To generate the PA signals efficiently, the thermal and stress confinements should be satisfied. These requirements mean that the pulse width of the laser should be short enough; and in order to attain enough SNR, the laser energy per pulse should be large enough. Meanwhile, utilizing LD aims at decreasing the volume and cost of the imaging system potentially for point-of-care testing. Thus, such a short and high-energy pulse is a challenge during the development of a laser system.

**Figure 1:**
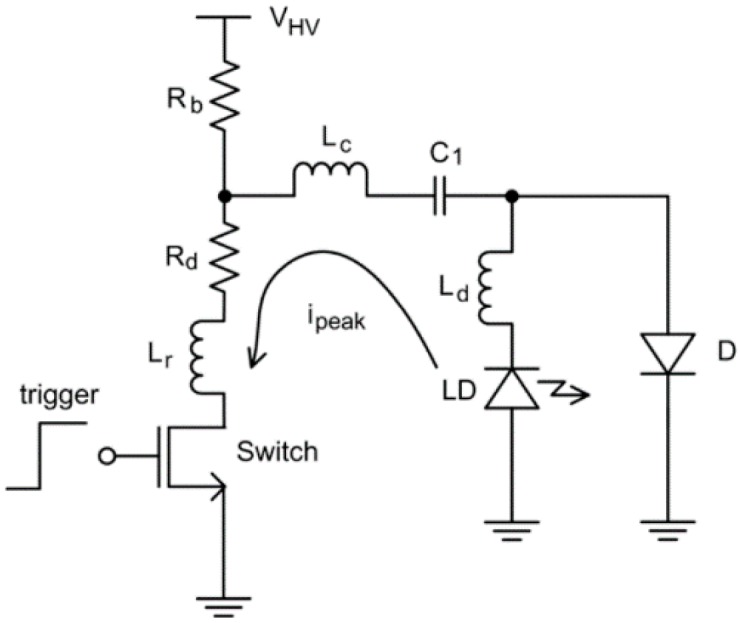
Structure of the typical laser diode driver. Reproduced with permission from Ref. [29].

From the perspective of the laser driver circuit, the typical driver circuit shows as in Figure 1. The operation principle of this circuit is based on the charge and discharge of the capacitor for pulse generation. The pulse current will flow through from LD to the switch device as illustrated by the arrow in Figure 1. In order to attain the minimized pulse width and maximize the peak current, the parasitic inductance (L_r_, Lc and L_d_) should be as small as possible [29]. Additionally, these kind of circuits are not able to modulate the pulse width since the time constant of the circuit is fixed. To our best knowledge, the ultra-compact pulse laser driver is reported in Reference [24], which has the size of 40 × 50 mm^2^, up to 10 kHz of repetition and the energy of 1.7 mJ per pulse within 40 ns width.

### 2.1. Short-Pulse Modulation Based on Laser Diode

There are many literatures on PA imaging based on laser diode by short-pulse modulation. Here, we will introduce some of typical and recent studies.

**Figure 2:**
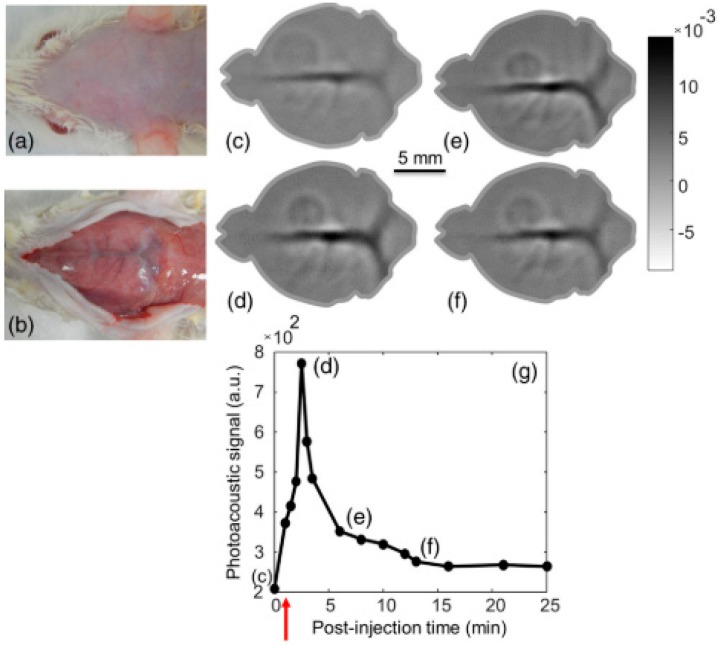
In vivo PLD-PAT brain imaging and pharmacokinetics of ICG intravenously injected in to rat brain: Photograph of rat brain before (**a**) and after (**b**) removing the scalp. In vivo brain images at different scan times (**c**) 0 s, (**d**) 2 min, (**e**) 6 min, and (**f**) 13 min. (**g**) Graph shows the quantification of ICG signal in the superior SS during 25 min following injection. The red arrow indicates the ICG injection point. Reproduced with permission from Ref. [30].

Dynamic in vivo photoacoustic imaging is a significant research area, which can monitor the blood velocity, oxygen saturation and circulating tumor to diagnose the processing of disease. Small animals play a significant role in preclinical studies and effectively reveal the experiment results. In Reference [30], the dynamic in vivo imaging in a mouse brain was achieved by using the pulsed laser diode as an illumination source. This system utilizes an 803 nm pulsed laser diode and a fast-scanning single-element ultrasound transducer. The repetition rate of the pulsed laser is about 7 kHz and its cross-sectional imaging time is about 5 s with a signal-to-noise ratio of about 48 dB. The spatial resolution is about 185 μm for 5 MHz transducers. The demonstration of dynamic imaging was performed by monitoring the uptake and clearance process of indocyanine green (ICG) in the cortical region of a mouse brain, which is shown in Figure 2. It is not very clear to show the deduction of ICG from the imaging results in Figure 2d–f, but from Figure 2g, the amplitude of the PA signal is decreasing apparently from point (d) to point (e). Additionally, as mentioned in Reference [30], the lateral resolution of this system could be further improved by using multiple ultrasound transducers.

**Figure 3:**
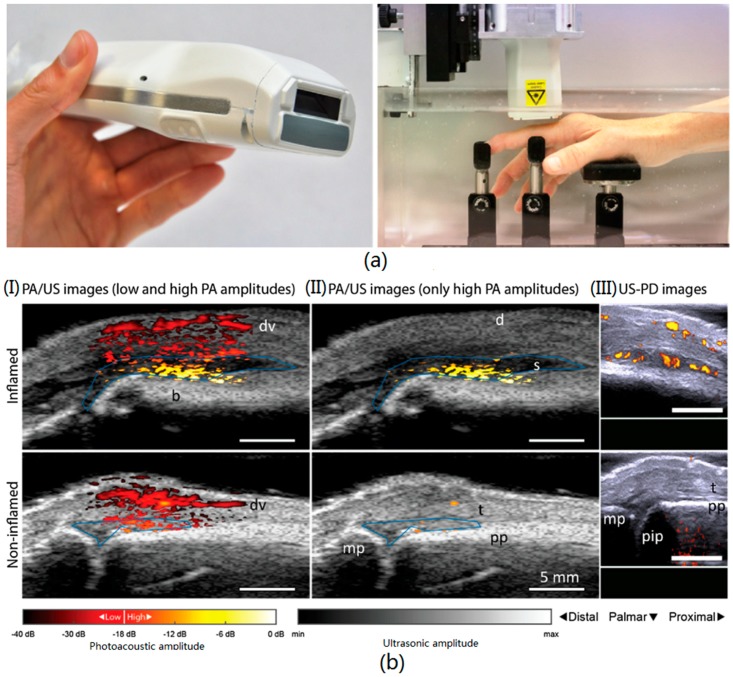
The imaging setup (**a**) and PA/US and US/PD images of an inflamed (upper row) and non-inflamed contra-lateral joint (bottom row) of an RA patient (**b**). PA/US images in (I) show a difference in color between inflamed and non-inflamed, corresponding to an increase in amplitude levels. When discarding low PA amplitudes in (II), only features in the inflamed joint are visible. Corresponding US-PD images are shown in (III). The blue line in the PA/US images indicates the ROI used for quantification of PA features in the synovial space. The 0 dB level is the maximum PA amplitude from the inflamed joint. d = dermis; dv = dorsal vein; pp = proximal phalanx; pip = proximal interphalangeal joint; mp = middle phalanx; s = synovium; t = extensor tendon. Reproduced with permission from Ref. [31].

The above imaging system verify the possibility of dynamic imaging in vivo. Moreover, the feasibility of diagnosing some diseases such as synovitis is also demonstrated [31]. The image system is shown in Figure 3a, which is a handheld probe integrated with a laser diode source, laser driver, ultrasound transducers and a prism [32]. The laser source in this research is at 808 nm wavelength. It can provide 1.3 mJ per pulse with 90 ns duration and its repetition rate can be up to 10 kHz. In Reference [31], it shows multimodality photoacoustic and ultrasound imaging of synovitis in finger joints. The clinically evident synovitis is detected by comparing the inflamed and non-inflamed proximal interphalangeal joints, which is shown in Figure 3b. The clinical study with a low-cost and compact photoacoustic and ultrasound probe is firstly demonstrated. This handheld probe is also used in the flow imaging system, which can achieve quantitative flow velocities from 12 to 75 mm/s [33]. Such a compact solution paves the way for clinical investigation of a photoacoustic imaging system based on economical pulse laser diode.

**Figure 4:**
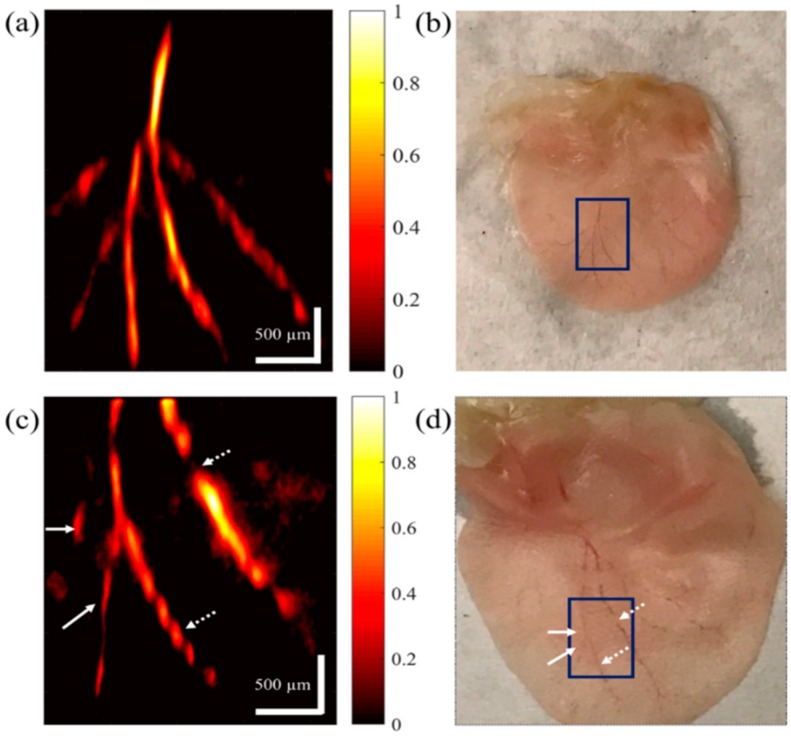
(**a**,**c**) PAM image of the vasculature on a mouse ear ex vivo. The color bars represent normalized PA amplitude. (**b**,**d**) Photograph of the mouse ear in (**a**,**c**), respectively. Reproduced with permission from Ref. [34].

The low output energy of the pulse diode causes many times the averaging signal required to improve the SNR of PA signals, which will limit the clinical application of the pulsed laser diode since the imaging speed is not enough and the imaging penetration will be significantly limited. Thus, improving the energy is of paramount importance. The proposed PAM system reported in Reference [34] utilizes the laser source that can output 325 W peak power pulse within 50 ns. It is not necessary for averaging during data acquisition in this system. Therefore, the imaging speed can be improved significantly, which reaches ~370 A-lines per second. Moreover, there is no need for mechanical scanning of the sample since two galvanometer scanning mirrors serve as the two-dimensional scanning of the beam through an aspheric focusing lens. The mouse ear ex vivo imaging shown in Figure 4 verified the feasibility of this system. Another method to improve output energy is to combine several laser diodes [25].

In addition to the above-mentioned reports, there are also many other interesting studies on photoacoustic imaging based on pulsed laser diode. Imaging the custom-made phantoms such as blood vessel phantoms or other tissue-mimicking phantoms are developed by many imaging systems [35,36,37,38,39,40,41,42]. For ex vivo imaging, several imaging systems are tested on chicken breast, mouse ear and porcine ovary etc. [43,44,45,46,47,48,49,50]. For in vivo imaging, blood vessels [26,51,52], hemoglobin monitor [53] and animal brain [54] imaging systems are also studied based on pulsed laser diodes. For photoacoustic sensing based on such economical laser sources, some systems are developed for gas detection [55,56].

Last but not least, it should be noted that laser diode has two types. One is the pulsed laser diode and another one is the continuous laser diode. Most photoacoustic imaging systems use the pulsed laser diode. Recently, the continuous laser diode is verified for its feasibility of working at pulsed mode by overdriving while avoiding the catastrophic optical damage and thermal damage [57]. It can be a complementary solution of the pulsed laser diode since the continuous laser diode has a broader available wavelength.

### 2.2. Short-Pulse Modulation Based on LEDs

**Figure 5:**
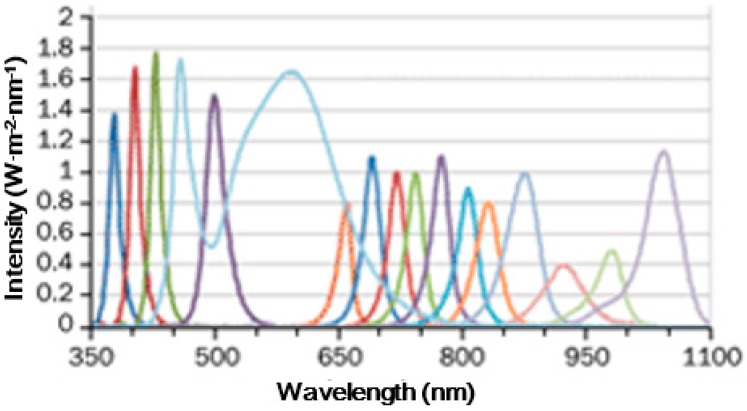
Individual spectra of 23 LEDs. Reproduced with permission from Ref. [63].

By overdriving the high power LEDs beyond their continuous current damage threshold, LEDs can be another alternative pulsed light emission source, which are more economical than LDs and less sensitive to static electricity [58]. Theoretically, both LEDs and LDs are semiconductor devices, which means their driver circuits are similar [58,59,60]. The fabrication process and technology of LEDs allow multi-wavelength in a single LED [61]. The multi-wavelength LEDs can be controlled to an output-specific wavelength by the electronic signals, which is an attractive characteristic in multi-wavelength PA imaging. The multi-wavelength characteristic of LED can significantly reduce the size of the illumination system since it is not necessary to combine several LDs in different wavelengths. However, the single-wavelength light intensity from multi-wavelength LEDs will decrease because the total chip area is constant, which is critical in PA imaging. Moreover, the efficiency of delivering the light power of LEDs from optical fiber is very low (10~20%), which means the higher power LEDs are needed to provide enough light density on the imaging object [62]. This will increase the volume of LEDs illumination system. The different principle of light emission between LDs and LEDs leads to the broader spectrum in the emission wavelength from LEDs shown in Figure 5 [61]. According to the temperature sensitive characteristics of semiconductor devices, the emission wavelength may shift considerably as the operation temperature increases [58]. This feature may influence the accuracy of PA imaging results. It should be noticed that the low prices of LEDs enable it to still be attractive in PA imaging.

In the field of PA imaging and sensing based on short-pulsed modulation, gas monitoring, phantom imaging and in vivo imaging were demonstrated.

Gas monitoring systems were developed in the early stage of LEDs. Lay-Ekuakille et al. developed a multi-gas sensing system for estimating the concentration of anaesthetic gas and nitrous oxide (N_2_O) in hospital to protect the faculties and patients [64]. Based on 4–4.5 μm LEDs, this system detects an attention threshold level of gas in a rough range. Other gas detection such as ozone by blue LEDs with 460 nm and water vapor by IR LEDs with 1450 nm were reported in Reference [65,66], respectively.

**Figure 6:**
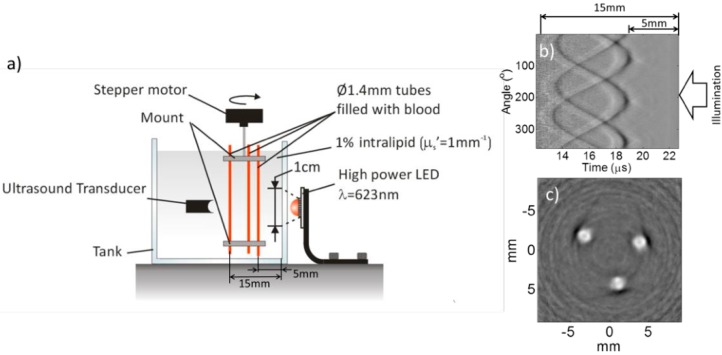
(**a**) Photoacoustic imaging setup. (**b**) Time-resolved photoacoustic signals of three 1.4 mm tubes filled with human blood (35% haematocrit) and immersed in 1% Intralipid (μ_s_′ = 1 mm^−1^), (**c**) reconstructed photoacoustic image. P = 9 µJ, N = 5000. Reproduced with permission from Ref. [27].

**Figure 7:**
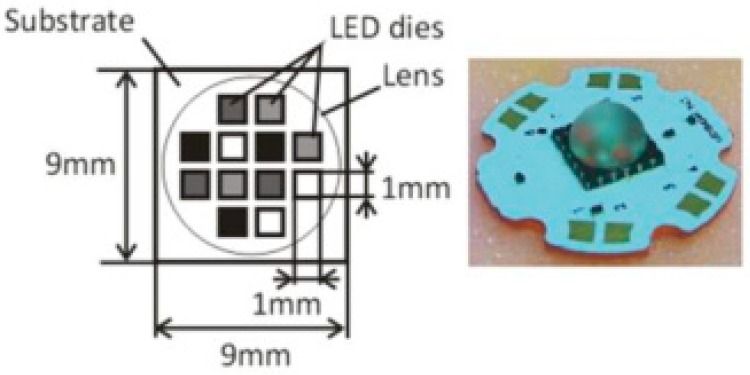
Schematic and photograph of the four wavelength LED (460, 530, 590, and 620 nm). Reproduced with permission from Ref. [27].

To image a tissue mimicking phantom, a 623 nm LED was overdriven at 50 A which is 20 times larger than its nominal current in Reference [27]. Figure 6a shows the setup of the PAT imaging system comprising of three tubes filled with human blood. The energy per pulse is 9 μJ with 500 Hz repetition rate. The duty cycle is well below 1% to prevent the LED damage. Figure 6b is the reconstructed image of detected time resolved PA signals as a function of scan angle. It shows that the penetration depth of this system can be 1.5 cm. Figure 6c shows the reconstructed PA imaging of three tubes. The diameters of the tubes in this image is within 0.1 mm, which is close to the real values. Moreover, this work has utilized a compact four wavelength LED, shown in Figure 7, to implement two excitation strategies to increase SNR. One is to use all four wavelengths simultaneously to design a Wiener filter, and another one is to use the extended Golay coded excitation. The latter excitation method shows the potential for faster data acquisition. The multi-wavelength imaging based on several single-wavelength LEDs is described in Reference [67], which illustrates the color imaging system.

**Figure 8:**
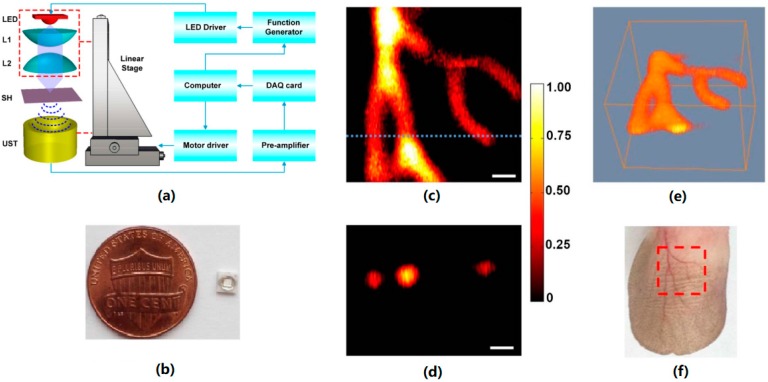
In vivo photoacoustic imaging of mouse ear vasculature. (**a**) LED-PAM system. L1, L2. Lens; SH, sample holder; UST, ultrasound transducer. (**b**) Photograph of LED. (**c**) Maximum amplitude projection (MAP) image. (**d**) Cross-sectional imaging. (**e**) 3D volumetric image. (**f**) Photograph of mouse ear. All the scale bars indicate 0.5 mm in length. Reproduced with permission from Ref. [60].

Xianjing Dai et al. demonstrated the in vivo imaging of vasculature based on LEDs [60]. A 1.2 W 405 nm LED with 200 ns pulses and repetition rate of 40 kHz was used to excite the photoacoustic signals in the PAM imaging system (shown in Figure 8a). Figure 8b shows an ultra-compact LED compared with a coin. Figure 8c,e are the maximum amplitude projection image and 3D volumetric image under the dotted red square in 8f, respectively. The dotted red square is the scanning area of 4 mm × 4 mm. Figure 8d is the cross-sectional image along the dotted line in 8c. The signal of each scanning point averaged 4000 times and the imaging speed is 10 A-lines per second. This study shows the potential of a portable handheld photoacoustic imaging device based on LEDs.

**Figure 9:**
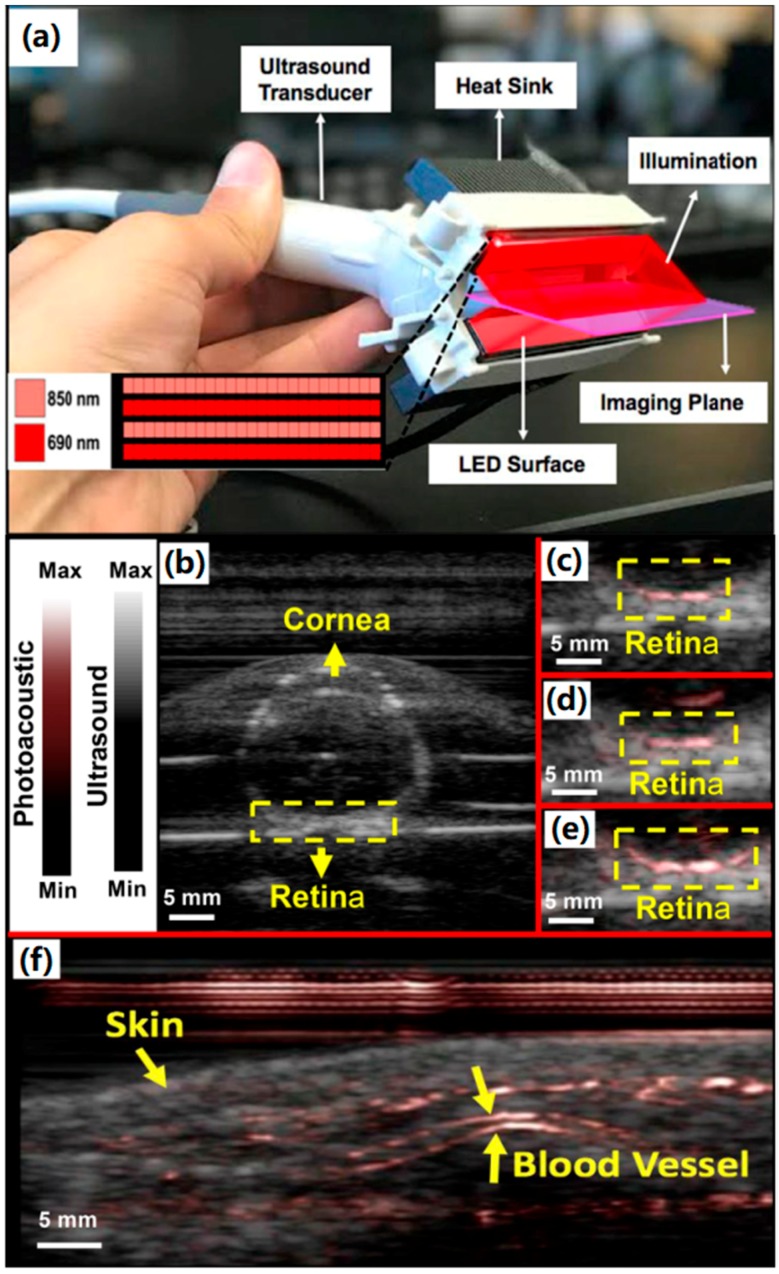
(**a**) PLED-PAI probe with imaging plane and illumination source are shown schematically. LED array design is also shown in the inset—there were alternating rows of LEDs with different wavelengths. (**b**–**e**) Evaluation of PLED-PAI on rabbit eye. (**b**) B-mode ultrasound image when fresh enucleated rabbit eye was embedded in 1% agar. (**c**) B-mode photoacoustic/ultrasound image of rabbit eye using 690 nm (**d**) B-mode Photoacoustic/ultrasound image of rabbit eye using 850 nm. (**e**) B-mode photoacoustic/ultrasound image when both 690 and 850 nm are used at the same time. Retinal vessels are imaged in a depth of 2 cm. (**f**) Photoacoustic image of skin and vasculature. Skin and blood vessel are shown using yellow arrows. Reproduced with permission from Ref. [68].

More recently, a portable light emitting diode—based photoacoustic imaging (PLED-PAI) system with a handheld probe was developed [68]. Figure 9a is the handheld probe in this dual modality (ultrasound/photoacoustic) imaging system. It integrates with 2 LED arrays and 128 elements of an ultrasound transducer. Each LED array has four rows of 36 single embedded LEDs. The emitted pulse width allows changing from 50 ns to 150 ns with a 5 ns step size and the repetition rate can be tuned between 1 kHz, 2 kHz, 3 kHz and 4 kHz. This system has the frame rates of 30 Hz, 15 Hz, 10 Hz, 6 Hz, 3 Hz, 1.5 Hz, 0.6 Hz, 0.3 Hz and 0.15 Hz, which depends on the choice of repetition rates of LEDs. The axial resolution is 268 μm and the lateral resolution is between 550 μm and 590 μm under the 70 ns pulse width. The penetration depth can be 3.2 cm when detecting a pencil lead inside chicken breast with a frame rate of 15 Hz. The dual modality imaging of a fresh enucleated rabbit eye illustrates the clinical utility of PLED-PAI, which is shown in Figure 8b–e. The yellow dotted box shows clearly the retinal vessels in 2 cm depth. Figure 8f is the imaging result of a human wrist, which shows the skin and vessels by yellow arrows.

**Figure 10:**
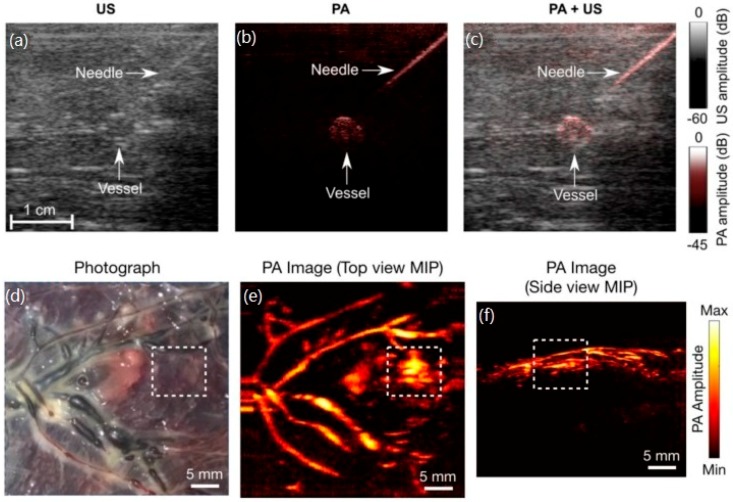
(**a**–**c**) Photoacoustic and ultrasound (US) imaging of a needle inserted towards a vessel mimicking phantom. (**a**) US image; (**b**) PA image at 850nm; (**c**) PA + US image overlay. (**d**–**f**) Human placental vasculature imaging. Photograph (**d**) and PA images (**e**,**f**) of a portion of the human placenta. Reproduced with permission from Refs. [69,70].

Furthermore, this portable PA imaging system has been exploited for accurate visualization of clinical metal needles [69] and human placental vasculature imaging [70]. The visualization of clinical metal needles is critical in guiding minimally invasive procedures, and PA imaging has shown promising potential in this field. Wenfeng Xia et al. have developed a combined handheld PA and ultrasound imaging system for real-time visualization of clinical metal needles. In this system, the needle is inserted into chicken breast tissue ex vivo and is visible with PA imaging at depths of up to 2 cm. The SNR of both US images and PA images decreases as the needle insertion angle increased. Figure 10a–c briefly show the imaging results of this system and a supplementary video is provided in Reference [69]. From Figure 10a–c, it can be seen that the visibility of the needle and vessel was low in ultrasound images but it is high in PA images and dual modality images. The human placental vasculature imaging based on such a handheld probe has the potential to be the guidance in minimally invasive fetal interventions, such as twin-two-twin transfusion syndrome (TTTS), to optimize the treatment outcomes. The preliminary study in Reference [70] demonstrated the feasibility of real-time PA/US imaging of the human placenta. Figure 10d is the photograph of human placenta whose facial vasculatures are visible in PA images (Figure 10e,f), which means the anastomosing vessels may be identified in TTTS. However, the clinical application of guiding TTTS based on PA imaging requires us to develop a miniature probe that is small enough to be delivered through the working channel of a fetoscope. These studies and such handheld imaging systems make a big step towards the clinical applications based on LEDs.

### 2.3. Long-Pulse Modulation and Quasi-CW Modulation

**Figure 11:**
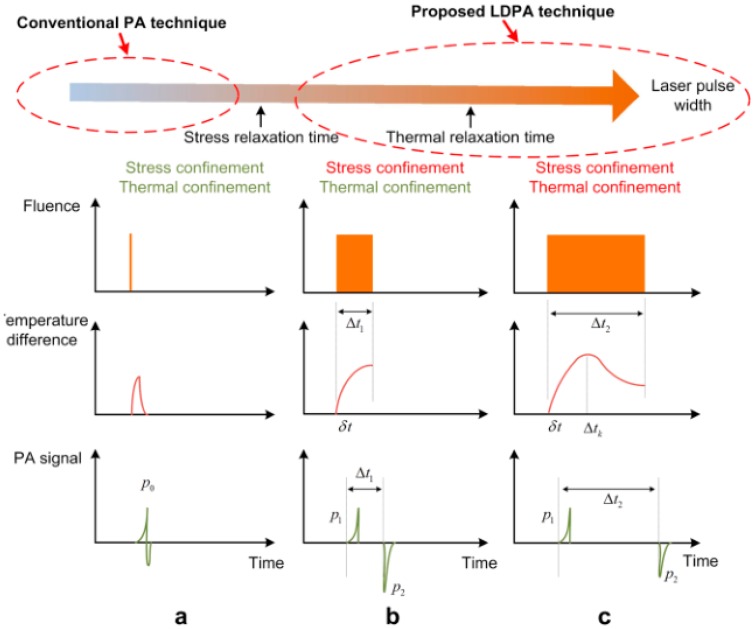
The fluence pattern, temperature change and PA signal waveform of (**a**) conventional short laser pulse-induced PA effect with both stress and thermal confinements satisfied; (**b**) long laser pulse induced dual PA nonlinear effect with only thermal confinement satisfied, and (**c**) even longer laser pulse without thermal confinement. Reproduced with permission from Ref. [71].

Long-pulse modulation and quasi-CW modulation can induce a photoacoustic nonlinear effect. The former is a novel method to induce dual PA signals by using the long pulse without satisfying the stress confinement [71]. Generally, a PA signal consists of a positive waveform and a negative waveform with the satisfaction of stress confinement and thermal confinement as shown in Figure 11a. Due to the pulse width longer than the stress of the confinement time, a PA signal will be separated into two parts, the positive one and the negative one as shown in Figure 11b. The positive signal and negative signal are induced by the sharp rising and falling edges of the square laser pulse, respectively. Meanwhile, the absolute amplitude of the second signal is larger than that of the first signal since the heat accumulation is greater than heat diffusion. With the increase of pulse width, if the heat accumulation is smaller than the heat diffusion, the second signal will drop back to the normal level shown in Figure 11c. On the contrary, if the heat accumulation equals to the heat diffusion, the second signal will be saturated. The theoretical analysis is derived in Reference [71]. This long laser pulse-induced dual PA (LDPA) nonlinear effect can be observed with low-power laser diode and has much broader application compared with those that only happen when laser fluence exceeds a threshold value [72,73].

**Figure 12:**
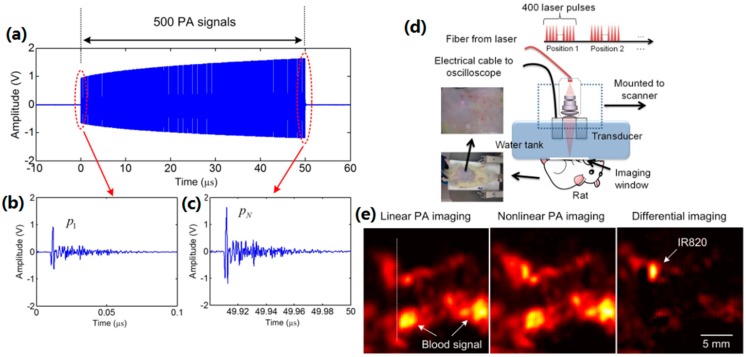
(**a**) A typical 500 PA signal, (**b**) the first linear PA signal, and (**c**) the last nonlinear PA signal. (**d**) In vivo experimental setup based on the LDPA technique by quasi-CW laser excitation. (**e**) The reconstructed linear PA, nonlinear PA and differential imaging results of a rat with subcutaneous injection of IR820 in the abdomen. Strong PA signals from high-absorptive blood background are shown in the linear and nonlinear PA images. By subtracting, the signal from the IR820 pops up with linear background suppression. Reproduced with permission from Ref. [71].

This nonlinear effect, based on the relationship between heat accumulation and diffusion, can also be observed at quasi-CW pulses excitation when the pulse-pulse interval is smaller than thermal relaxation time. Figure 12a shows the increasing amplitude of PA signals induced by the consecutive pulses. Comparing with Figure 12b,c, the last nonlinear PA signal is much greater than the first linear PA signal. This kind of characteristic can improve the imaging contrast by differential imaging. Figure 12d is a PAM in vivo imaging setup based on the quasi-CW excitation. The abdomen region of a 7-week-old rat was injected with a kind of NIR organic dye—IR-820 which has quite a low thermal conductivity and a good thermal confinement property. The imaging results are shown in Figure 12e. Due to the existence of the blood spot at the surface of the abdomen skin, the linear PA imaging shows the strong blood signals and weak IR-820 signals. In nonlinear PA imaging, the IR-820 signals are stronger due to the heat accumulation. Furthermore, the differential imaging can easily identify the position of IR-820. The image contrast can also be improved from 0.49 (linear PA imaging) to 1.7 (nonlinear PA imaging) and 12.3 (differential imaging).

### 2.4. Coded Excitation

Although the LDs or LEDs can serve as a compact and economical light source for the PA system, the large number of averaging of the received signals is required to improve the SNR, which will limit the imaging speed. Coded excitation is an alternative technique to induce PA signals and improve SNR instead of data averaging. Moreover, under the conventional averaging method, the repetition rate of lasers will be limited by the time of flight of the acoustic signal. The coded excitation can be used to overcome this limitation. In this method, the consecutive coded sequence is transmitted from the light source and the corresponding induced PA signals are properly filtered and decoded to generate the desired PA signals [74]. The desired PA signals exhibit a higher mainlobe-to-sidelobe ratio leading to the improvement of the SNR.

**Figure 13:**
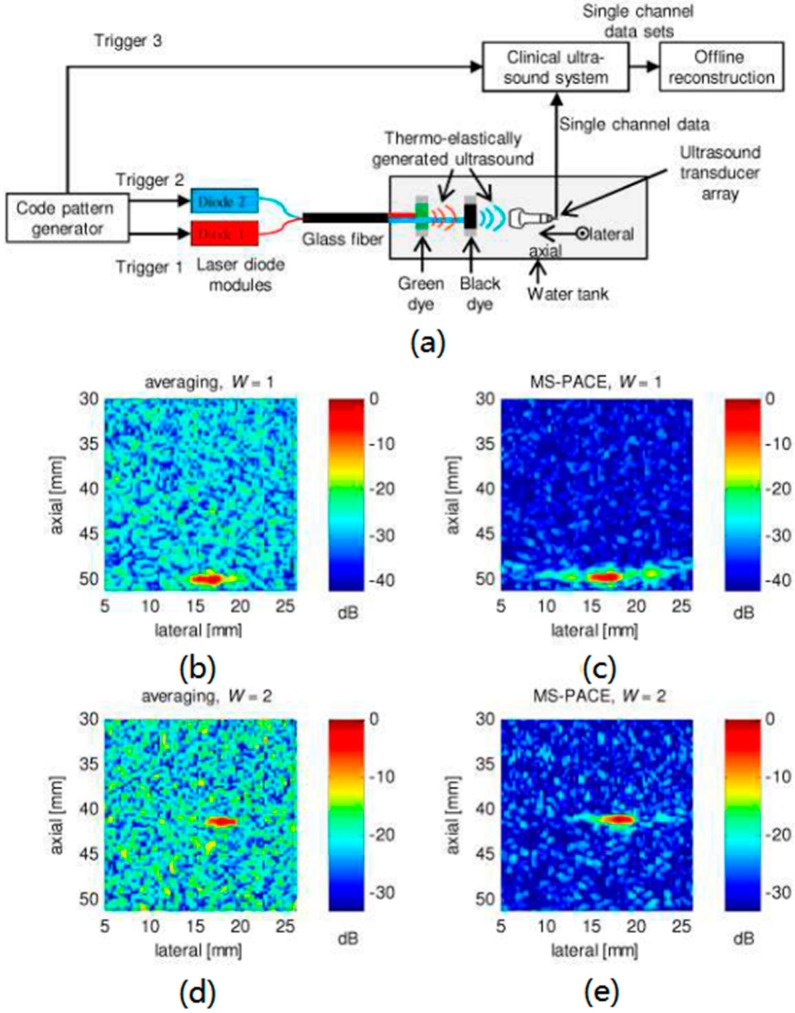
(**a**) Experimental setup for MS-PACE. (**b**–**e**) Comparison of MS-PACE and time equivalent averaging for both excitation wavelengths. (**b**) PA image for averaging as many acquisitions as possible during the coding procedure for W = 1, termed time-equivalent averaging. (**c**) PA image using MS-PACE for W = 1. (**d**) PA image using time-equivalent averaging for W = 2. (**e**) PA image using MS-PACE for W = 2. Reproduced with permission from Ref. [76].

Golay codes is one of the coded excitation schemes. There are two main advantages in Golay codes [75]. One, is the improvement of SNR of PA signals as a function of $\sqrt{N}$ where N denotes the code length. Another one is the simultaneous acquirement of the PA signals at multi-wavelength by exploiting the different sets of orthogonal codes. Golay codes consist of two biphase orthogonal codes but the light source is unable to emit the negative signals, so that every biphase codes will be split up into two unipolar parts that contain positive parts and negative parts, respectively. Thus, the single-wavelength Golay codes excitation consist of four excitation sequences. The elimination of sidelobes of PA signals benefits from the complementary code pairs. In Reference [76], the multispectral photoacoustic coded excitation (MS-PACE) based on unipolar orthogonal Golay codes was discussed. It contains eight unipolar coded sequences and simultaneously acquires the PA data sets from two irradiation wavelengths and then separates them. In Figure 13a, a 652 nm custom-made high power quasi-continuous laser diode bar (diode 1) with a maximum optical power of 100 W and an 808 nm laser diode bar with 225 W were used in this experimental setup for MS-PACE. The light from diode 1 was mostly absorbed by the green dye so that the PA response from the black dye is mainly related to the light from diode 2. For these experiments, the code length is 512 bit and the light repetition rate is 500 kHz. The total time to send the codes is 4.2 ms, which can obtain 63 waveforms per wavelength for the conventional averaging method. Consequently, the imaging results by coded excitation (Figure 13c,e) are compared with that by 63 averaging times (Figure 13b,d). The results show that the noise floor of the averaging method is approximately 9 dB higher than that of the MS-PACE method for each wavelength, which corresponds to the calculated coding gain (SNR improvement) of roughly 9 dB. Furthermore, there is no crosstalk and correlation sidelobes in the images based on MS-PACE. In addition to Golay code, another coding strategy for multiple wavelength, pseudorandom codes, is discussed in References [77,78,79], whose coding gain exceeds that of orthogonal Golay codes for finite code lengths.

The Legendre sequences have simpler coding procedure than unipolar Golay codes since it only consists of two unipolar sequences [80]. It can slightly exceed the gain of Golay codes for finite code sending time, and the range sidelobes remain invisible if the code length is sufficiently large. However, it is not suitable for multi-wavelength imaging since the multiple signals cannot be separated. Moreover, the further simple coding procedure is achieved in periodically perfect sequences (PPS), as only a single sequence needs to be repeated continuously [81]. PPS is suitable for continuous acquisition without the artifact and the performance is better than the above coding strategies.

Some modulation techniques can also be used in coded excitation such as frequency modulation [82] and pulse position modulation (PPM) [83]. The frequency modulation is to tune the interval between two adjacent laser pulses by changing the period of pules. The PPM is to tune the pulses position at the same period. Both two techniques will induce the sidelobes because they consist of only a single code sequence. However, the coding gain of both methods performs well and exceeds that of previous code strategies. PPM codes are suitable for short code applications since the performance will drop for long codes.

## 3. Continuous Modulation

Compared with the pulsed excitation that is used in time-domain photoacoustic imaging, the continuous modulation is mostly used in the frequency-domain (FD) photoacoustic (FDPA) imaging, which employs light intensity modulation to generate the PA signals [84]. It could be considered as another kind of code excitation method [85]. It is more economical than pulsed modulation because it uses the low-cost continuous laser diodes with a specific modulation scheme. In addition, the SNR will be remedied by employing the cross-correlation operation [86].

### 3.1. Chirp Modulation

**Figure 14:**
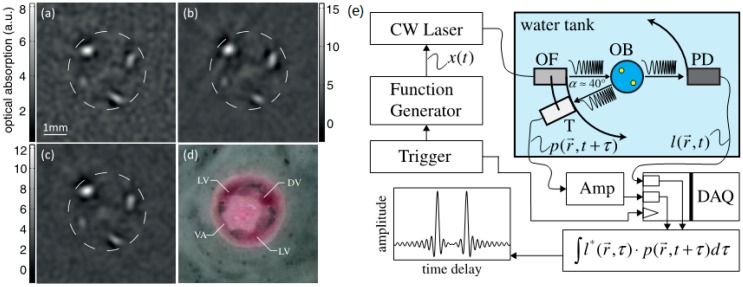
(**a**) FD-optoacoustic tomography (FD-OAT) in vivo mouse tail image without ICG, (**b**) FD-OAT tail image during ICG injection, (**c**) tail image ~10 min after initial ICG injection, (**d**) cryoslice of the mouse (LV, lateral caudal veins; DV, dorsal caudal vein; VA, ventral caudal artery; dashed circle represents approximate tail surface), (**e**) schematic diagram of the frequency domain optoacoustic tomography system. An optical fiber (OF) guides the laser chirps onto the object (OB). Signals from the photodetector (PD) and the transducer (T) are acquired simultaneously by the data acquisition (DAQ) and afterwards are cross-correlated. Reproduced with permission from Ref. [90].

The chirp modulation is implemented by changing the excitation frequency linearly, which is a typical modulation method in frequency domain [87,88,89]. The correlation processing (matched filter compression) and heterodyne mixing with coherent detection are the two main processing approaches for FD signals [84]. The FD imaging has been implemented in x-y scan mode, whereas Stephan Kellnberger et al. proposed a novel scan mode that requires multiple projections (angles) and mathematical inversion [90]. In Figure 14e, the optical fiber (OF) and transducer (T) are rotating simultaneously by the step sizes of 2°, resulting in 180 projections and ~10 min for a whole image. A 808 nm CW laser diode with a peak power of 500 mW and a focused transducer with 3.5 MHz central frequency and 76% bandwidth were used. The frequency sweeps from 1 to 5 MHz with a duration of 1 ms. To validate the ability of tissue imaging in vivo by this FD optoacoustic tomography setup, the mouse tail was imaged at a height of ~4 cm from the distal end with the injection of Indocyanine green (ICG). The lateral caudal veins, dorsal vein, and the ventral caudal artery are visible in Figure 14a. The tail in Figure 14b was injected with ICG and after ~10 min its imaging result was record in Figure 14c to investigate the dynamic change of ICG in the tail. The comparison of these two figures shows that the light absorption intensity reduces in Figure 14c as the ICG fading. The proposed scan mode can realize diffraction-limited resolution through retrieving much higher effective apertures. This potentially leads to superior imaging performance and SNR.

In PA imaging, the measurement of hemoglobin oxygen saturation is one of the significant applications [91]. The laser fluence is assumed to be known in the tissue when uses two wavelengths to measure the hemoglobin oxygen saturation. In reality, the fluence is unknown because the optical scatter is not wavelength-independent, so this assumption will cause some error [92]. Bahman Lashkari et al. proposed a method which employs the phase of the FD photoacoustic signals to measure the absorption coefficient and quantitatively probe the hemoglobin oxygen saturation without the influence of fluence [92]. However, the prerequisite for using this method is to know the chromophore geometry. In Fourier domain, the generated PA signals will be affected by the fluence, and the function of the corresponding cross-correlation signals contains the fluence parts, while the phase of cross-correlation signals does not. That yields the absorption coefficient of the chromophore in an analytical form, which can be used to attain the absorption coefficient quantitatively. It also shows that only one wavelength is required. Through this method, the error of phase measurement of oxygen saturation was 5.3% while the amplitude measurement error was 12.2%.

**Figure 15:**
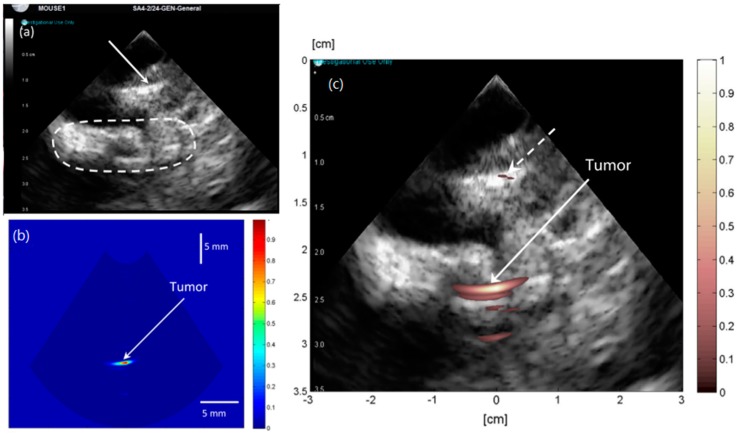
(**a**) Pure ultrasound image showing the bottom edge of the plastic seat (arrow) and region of interest (oval); (**b**) improved post-amplification PA image of tumor in right thigh of nude mouse. (**c**) Improved filtered PA image superimposed on the pure ultrasound image of the right thigh of the mouse. Reproduced with permission from Ref. [94].

To provide sufficient diagnostic information, combining different imaging modalities is necessary, which can attain the complementary information. Pervious investigations have combined FD photoacoustic imaging and ultrasound imaging for intravascular applications [93]. This paper employed 1 W 1210 nm continuous LD with the intensity-modulated chirp frequency of 4–12 MHz. The agar vessel phantom with graphite and lipid targets was employed to validate the feasibility of this dual modality imaging system. Moreover, such dual-modality imaging systems make sense for tumor detection [94]. For this literature, the linear frequency-modulated chirp signals (0.5 MHz to 4 MHz, 1 ms duration) was produced by an 808 nm LD with output power of 6 W, and the PA signals detection as well as the ultrasound imaging were implemented by a 64 element, 3.3 MHz phased array transducer. The imaging object was a mouse that was injected with a cultured human hypopharyngeal head and neck squamous cell carcinoma FaDu cells in the right thigh to cause the tumor to grow in the mouse. The results show that the ultrasound imaging cannot distinguish the tumor as the interference of the surrounding soft issue such as the dash line in Figure 15a. PA imaging is sensitive for tumor detection due to the increased blood flow in tumor, which can be seen in Figure 15b. Figure 15c is the co-registered image of Figure 15a,b, which provides complete diagnostic information for tumor detection. It indicates the relative position of the tumor compared with the other soft issue in the mouse thigh.

### 3.2. Sinusoidal Modulation

Driving the LDs or LEDs by sinusoidal modulation with single frequency to implement PA imaging is a special case of FDPA. In the case of sinusoidal excitation, SNR of the PA signals will be smaller than that of the pulsed mode, which has been discussed in Reference [95]. There are two main factors also presented in Reference [95] to remedy this difference. Firstly, because the noise amplitude is proportional to the square of the signal bandwidth, the narrowband signals generated by sinusoidal modulation could increase the SNR to some extent. Secondly, the transducer used in pulsed modulation mode is required to have a broad bandwidth, which will cause insertion loss because of the low electromechanical coupling coefficient of piezoelectric material or the high acoustic impedance. However, a resonant and low-loss transducer can be used in sinusoidal modulation mode, which can exhibit much higher detection sensitivity than wideband transducers.

**Figure 16:**
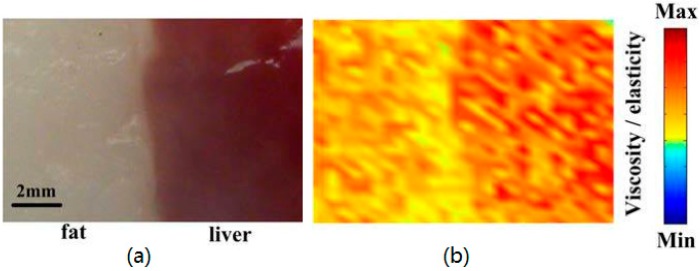
(**a**) Photograph of biological tissues (fat and liver). (**b**) Viscoelasticity distribution of the sample detected by PAVEI. Reproduced with permission from Ref. [96].

The characteristic of phase delay of PA signals induced by sinusoidal modulation can be exploited to achieve photoacoustic viscoelasticity imaging (PAVEI) since different viscoelasticity will lead to a different phase of the PA wave [96]. The thermal stress will change periodically when there is cyclical heating in the local region. The corresponding generated strain will also change periodically but will have a delay with the thermal stress due to the viscoelasticity of biological tissues. By establishing the relationship of the PA phase delay and the viscoelasticity of soft issue, the PAVEI of tissue could be implemented. An 808 nm CW laser was used with the modulation frequency of 50 kHz and a lock-in amplifier was used to calculate the phase delay between the dominant frequency PA wave and the reference signal. Figure 16b shows the PAVEI results in Reference [96], which shows that the viscoelasticity distribution of the sample can be mapped. This technique could possibly be applied to the detection of atherosclerotic plaque and skin tumor since the change in viscoelasticity could herald the diseases.

**Figure 17:**
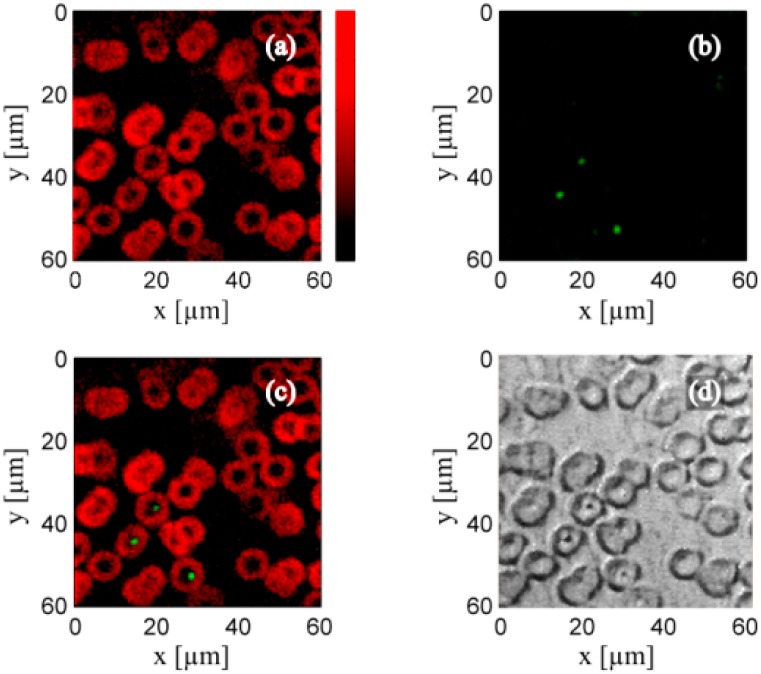
(**a**) OR-PAM image (**a**) and simultaneously obtained luminescence image (**b**) of human red blood cells. (**c**) Overlay of the PA and luminescence image. (**d**) Bright-field image of the same region. Reproduced with permission from Ref. [97].

As mentioned above, the FDPA imaging has combined with ultrasound imaging to provide the complementary information. For Sinusoidal modulation, it has combined with fluorescence microscopy to map the fluorescence position in blood smear [97]. In this imaging system, a continuous LD of 405 nm wavelength with an output power of 120 mW was modulated with the frequency of 10 MHz and the FWHM of the laser spot was ~750 nm. The shape of blood cell on blood smear is clear in PA imaging (Figure 17a) and some fluorescent spots can be seen in the luminescence image (Figure 17b). The overlay of these two results (Figure 17c) reveal that the fluorescent spot is from the blood cell. In general, the normal blood cell does not express the characteristics of fluorescence. The origin of the fluorescence is unknown. However, this system validates the important combination of these two imaging modalities as they can provide complementary information for each other.

**Figure 18:**
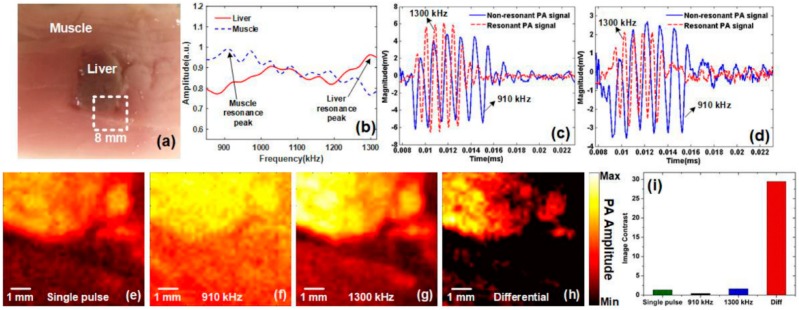
(**a**) Photograph of porcine tissue. (**b**) The PA resonance spectra of porcine muscle (blue dashed) and liver (red solid). (**c**) PA waveforms of liver at resonance (1300 kHz, dashed red line) and off-resonance (910 kHz, solid blue line). (**d**) PA waveforms of muscle at resonance (910 kHz, solid blue line) and off-resonance (1300 kHz, dashed red line). (**e**) Conventional single-pulse-induced PA image (**f**) PARI at muscle resonance (910 kHz). (**g**) PARI at liver resonance (1300 kHz). (**h**) Differential image. (**i**) Contrast comparison of Figure 5e–h. Reproduced with permission from Ref. [99].

Furthermore, the image contrast under sinusoidal modulation could be enhanced significantly due to the PA resonance effect [98]. The expression of acoustic pressure is the equation of motion of a forced harmonic oscillator with damping. Thus the amplitude of PA signals exists at a maximum value when the resonance phenomenon happens. Based on this resonance effect, a novel imaging modality, named photoacoustic resonance imaging (PARI), is proposed in Reference [99] and the great improvement of image contrast was demonstrated using tissue-mimicking phantom and ex vivo tissue. The ex vivo tissue shown in Figure 18a consists of the porcine muscle and liver issues of which resonance spectra are shown in Figure 18b. It can be seen that the highest amplitude of PA signals of porcine muscle and liver are at the frequency of 910 KHz and 1300 KHz, respectively. The following PA resonant imaging experiments regard these two frequencies as the resonance frequency of muscle and liver respectively. However, it should be noticed that from Figure 18b, the signal from the liver increases and that from the muscle decreases, which means that the resonance frequency could not be directly distinguished because there is no Lorentz-shape function in this image. Due to a lack of enough broad modulation frequency range in this paper, the resonance frequency may be distinguished if broadening the modulation frequency range. Figure 18c,d shows that the amplitude of PA signals at resonant modulation frequency will be higher than that at off-resonant modulation frequency. The imaging area is the white dotted square shown in Figure 18a. Figure 18e is the conventional single-pulse-induced PA imaging. The differential image (Figure 18h) is obtained by subtracting the liver resonance image (Figure 18g) from the muscle resonance image (Figure 18f). As shown in Figure 18i, the differential image shows a much higher image contrast than the other images.

### 3.3. Hybrid Modulation

**Figure 19:**
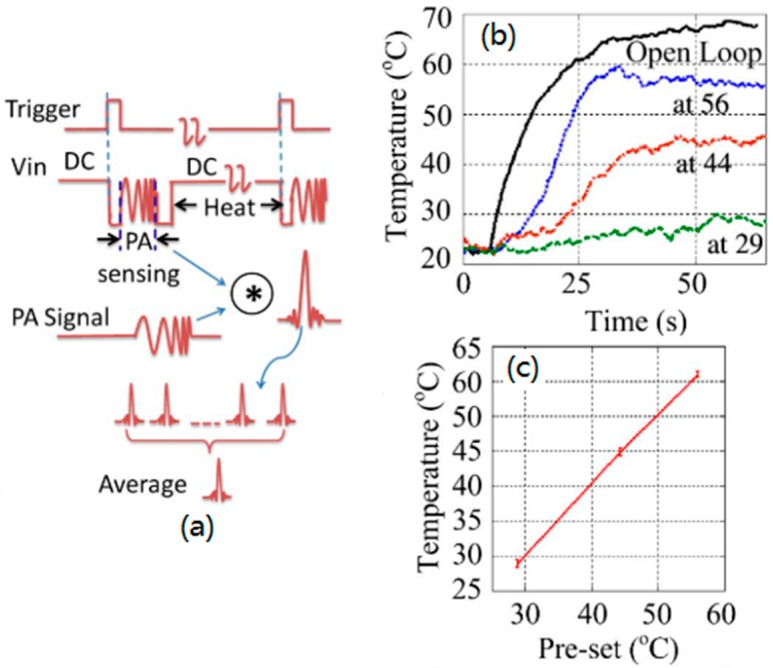
(**a**) Modulation scheme of the laser diode. (**b**) Self-temperature regulation with closed-loop pre-set values at 29 °C, 44 °C, and 56 °C, respectively. (**c**) Temperature-regulation accuracy analysis results. Reproduced with permission from Ref. [100].

The hybrid modulation combines different modulation types to excite a single laser source. To the best of our knowledge, there is an article to utilize the hybrid modulation for temperature monitoring and regulation in photothermal therapy [100]. This article discusses a laser-shared method to achieve temperature monitoring, regulation and photothermal therapy simultaneously. Figure 19a is the modulation scheme of the laser diode, which shows that the FDPA temperature measurement with chirp modulation and photothermal heating with constant laser intensity are alternating quickly in the time domain. To estimate the temperature increase according to the amplitude of PA signals, the PA signals were correlated with the reference signals and then averaged. A proportional-integral-derivative (PID) controller is adopted in the feedback loop to implement the self-temperature regulation. The acousto-optic modulator (AOM) is used to modulate the light into chirp pulse for PA imaging. The heating dose of the laser is controlled by a mirror driven by a motor. A 405 nm laser diode with a maximum power of 1 W is used and the phantom is a black wire with a radius of 1 mm. The chirp frequency sweeps from 0.5 to 1.5 MHz in PA temperature-measurement mode. Before measuring the temperature, this temperature measurement system is calibrated by using a K-type thermocouple. It can be seen from Figure 19b that the temperature can be maintained stably on the pre-set temperature except for the open loop photothermal therapy. Figure 19c shows that the accuracy of temperature regulation is 0.9 °C, and the accuracy of temperature measurement is 0.75 °C. This system can achieve ultrafast (4 KHz) temperature measurement. The accuracy improvement requires the enhancement of signals’ SNR that will be achieved by a longer chirp signal. Therefore, there is a tradeoff between the measurement speed and accuracy.

## 4. Discussion and Conclusions

The LDs and LEDs prompt the low-cost and compact PA sensing and imaging system. However, the weak SNR of the PA signals needs to be paid extra attention, due to the much low energy emitted by LDs or LEDs. To solve this challenge, firstly, the most direct method is to improve the output power of these semiconductor diodes to enhance the SNR. To the best of our best knowledge, the maximum output peak power of 325 W 905 nm laser diode with 50 ns pulsed duration was demonstrated to excite the PA signals with sufficient SNR (18 dB for imaging porcine ovary ex vivo) and no averaging was required [34]. Secondly, the most commanding and simple method to increase SNR is to average hundreds of PA signals at the same irradiation point, which will limit the imaging or detection speed. Thirdly, as mentioned above, the coded excitation could enhance the SNR through the correlation operation between the PA signals and reference signals, and the enhancement is proportional to the coded length. Moreover, the continuous modulation could facilitate the more low-cost imaging or sensing system since the required output power of the light source is much lower than the pulsed modulation. Fourthly, the contrast agent could be used to generate larger PA signal amplitude, such as gold and silver nanoparticles and gold nanorods [101,102,103,104,105,106,107,108,109]. Moreover, the double-walled carbon nanotubes could be used as nontoxic and biodegradable contrast agents [110]. Fifthly, some algorithm also could be exploited to improve the SNR such as the adaptive line enhancers algorithm [111]. Finally, other methods such as the empirical mode decomposition method and wavelet-based methods are widely investigated by many research groups [112,113].

To conclude, Table 1 is the summary of the different modulations with different light sources that have been discussed in this review. This paper has discussed the recent development of low-cost photoacoustic sensing and imaging systems based on LDs or LEDs according to different modulation schemes. Through the development of these systems, it can be considered that the clinical diagnosis and application of photoacoustic imaging requires that compact and affordable light sources replace bulky and expensive light sources. Fortunately, many prototype systems have validated the feasibility for specific functions such as hemoglobin oxygen saturation detection [92], in vivo small animal brain imaging [30], relative temperature detection [100], etc. Furthermore, the commercialized and portable PA imaging systems have been developed, by which the synovitis detection [31] and rabbit eye imaging [68] were achieved. It heralds a promising scenario for clinical application of PA imaging and sensing.

## Figures and Tables

**Table 1:** The summary of different modulation modes based on different light sources. PLDs, pulsed laser diodes; CW-LDs, continuous laser diodes.

Light Source	Modulation Mode	Advantages	Disadvantages	Application Example
PLDs	Single pulse	Simple excitation technique	Low SNR	Portable imaging device [31]
	Code	High speed and high SNR	Complicated code sequence	Multi-wavelength imaging [76]
	Quasi-CW	Provides another image contrast	Too much heating dose	Nonlinear PA imaging [71]
LEDs	Single pulse	Lower cost	Low fiber couple efficient	Portable imaging device [68]
	Code	High speed and high SNR		Multi-wavelength imaging [27]
CW-LDs	Chirp	High speed and high SNR	Complicated data processing	Dual modality imaging [94]
	Sinusoidal	High speed and higher SNR	No depth information	Viscoelasticity imaging [96]
	Hybrid	Real-time PA sensing	Extra equipment to modulate the light	PA sensing [100]
	Single pulse	Simple excitation technique	Low SNR	Human skin imaging [34]
